# Isolation of High-Quality Total RNA from Chinese Fir (*Cunninghamia lanceolata* (Lamb.) Hook)

**DOI:** 10.1371/journal.pone.0130234

**Published:** 2015-06-17

**Authors:** Zhihui Ma, Binlong Huang, Shanshan Xu, Yu Chen, Shubin Li, Sizu Lin

**Affiliations:** 1 College of Life Sciences, Fujian Agriculture and Forestry University, Fuzhou, China; 2 State Forestry Administration Engineering Research Center of Chinese Fir, Fuzhou, China; 3 College of Forestry, Fujian Agricultural and Forestry University, Fuzhou, China; Università della Calabria, ITALY

## Abstract

RNA isolation with RNA in a high quantity is a basic analytical method in plant genetics, molecular biology and related physiological investigations. To understand the genetic and molecular biology of Chinese fir, sufficient high-quality total RNA must be obtained for cDNA library construction and other downstream molecular applications. However, extracting RNA from Chinese fir is difficult and often requires the modification of existing protocols. Chinese fir tissues containing large amounts of polysaccharides and polyphenol compounds and are one of the most difficult plant tissues for RNA isolation. Therefore, we developed a simple method for extracting high-quality RNA from Chinese fir tissues. RNA isolations were performed within two hours, RNA quality was measured for yield and purity. Total RNA obtained from this procedure was successfully used for cDNA library construction, RT-PCR and transcriptome sequencing. It was proven that extracted RNA was intact and suitable for downstream molecular applications, including RT-PCR and qPCR, and other downstream molecular applications. Thus, this protocol represents a simple, efficient, and low-cost method.

## Introduction

Obtaining high-quality RNA in a large quantity is a preliminary step for many investigations in plant molecular biology such as reverse transcription polymerase chain reaction (RT-PCR), real-time fluorescent quantitation polymerase chain reaction (qPCR), cDNA synthesis and cDNA library construction, gene isolation, gene expression analysis, and next generation sequencing [1]. However, the extraction of total RNA from plant tissues rich in polyphenolics, polysaccharides, proteins and other secondary metabolites is often a time-consuming and tedious task [2]. Large amounts of these substances can interfere with the RNA isolation procedure. Polyphenolics bind irreversibly to RNA and proteins to form high-molecular-weight complexes, whereas polysaccharides coprecipitate with RNA in low ionic strength buffers [3]. Indeed, the presence of polyphenolics together with polysaccharides makes the extraction of high-quality RNA problematic. Therefore, it is necessary to develop an efficient method for template extraction of high-quality RNA from plant tissues rich in polysaccharides and polyphenolics for downstream molecular analysis.

To date, many published conventional RNA extraction methods, such as cetyltrimethylammonium bromide (CTAB), TRIzol Plus RNA Purification kit (Invitrogen, USA), RNeasy plant Mini kit (Qiagen, Germany) and other RNA reagent kits are currently available for extraction of RNA from plant tissues [4,5]. Each of the methods is specifically designed for specific plant species and the nature of the target tissue [5]. Some modified methods of CTAB have been used for extracting high-quality RNA from recalcitrant plants [6,7]. Huang and coworkers used the PureLink Plant RNA Reagent (Invitrogen, USA) to isolate total RNA of nine tissues of Chinese fir [8]. Chinese fir is one of the most important coniferous species, with high yield, excellent wood quality, versatile uses, and pest resistance. It is an evergreen coniferous tree primarily distributed in southern China and northern Vietnam [8].

However, extracting high-quality RNA from Chinese fir tissues is extremely difficult because Chinese fir is often rich in polyphenols, polysaccharides, proteins, lipids, and several other secondary metabolites that interfere with RNA extraction by degrading or coprecipitating with RNA. In addition, using an RNA isolation kit to extract the total RNA of Chinese fir tissues is expensive. Therefore, it is necessary to find an efficient, rapid, and reproducible method for isolating high-quality RNA from Chinese fir tissues.

In this study, we developed a simple, less expensive, and time-saving RNA extraction procedure for Chinese fir tissues (root and leaf) that efficiently yields high-quality RNA, modifying the method described by Chan et al [9]. High-quality RNA with no DNA contaminations was obtained through the protocol described here and is completely suitable for RT-PCR, qPCR and the construction of cDNA libraries.

## Materials and Methods

### Sampling

Fully expanded roots and leaves were sampled from Chinese fir (*Cunninghamia Lanceolata* (Lamb.) Hook) plants grown in greenhouses and frozen immediately in liquid nitrogen (This study did not involve endangered or protected species. These materials belonged to State Forestry Administration Engineering Research Center of Chinese Fir, China.). All samples were stored at -80°C before RNA extraction.

### Basic protocol

The following protocol was modified from the manufacturer’s provided instructions for effective use of the Plant RNA Isolation Mini Kit from Agilent Technologies. As indicated, all solutions were prepared with sterile RNase-free water, and all supplies and handling materials were RNase-free. This protocol is optimized to isolate total RNA from approximately 0.1 g of plant tissue. If the amount of plant tissue is increased, reagent volumes must be scaled appropriately.

### Solutions and reagents

SDS extraction buffer (SEB) (0.25 M, NaCl; 0.05 M, Tris-HCl (pH = 7.5); 0.02 M, EDTA; 1%, SDS) was modified from the protocol proposed by Chan et al [9] with the exception of the use of 4% (w/v) PVP (M.W.360000). In addition, 5 M NaCl, phenol:chloroform (v/v = 1:1) and 75% (v/v) ethanol were also used.

### Grinding the tissue

One of the critical points for obtaining a high yield in the extraction of genetic material is the grinding. It is essential to grind the tissue as finely as possible, maintaining samples as cold as possible during grinding to avoid degradation.

A high-throughput organization grinding machine (Scientz-192, Scientz, Ningbo, China) was used for grinding roots and leaves. Approximately 0.1 g of frozen roots and leaves were placed in 2 ml RNase/DNase-free tubes; then, two 5 mm sterilization steel balls were added into the same tube. Subsequently, the tubes containing the frozen tissue plus steel balls were put in the adapter to pre-cool with liquid nitrogen for 2 min and then shaken in the high-throughput organization grinding machine (Scientz-192) for 3 min at frequency 20 (1200 times/min) without buffer at low temperature.

### RNA isolation

RNA isolation was carried out based on a protocol proposed by Ni [10] for RNA extraction from Chinese fir following several major modifications:

1. 1 ml SEB and 50μl 2-mercaptoethanol (SEBM) were mixed sufficiently in a 1.5 ml RNase-free microcentrifuge tube.
2. 1 ml SEBM was added to pulverized tissue, which was mixed by brief vortexing or flicking the bottom of the tube until the sample was thoroughly resuspended. Then, the sample was incubated for 10 min at room temperature.
3. The mixture was centrifuged for 15 min at 13000×g in a microcentrifuge at 4°C. The supernatant was transferred (~600μl) to a new 1.5 ml RNase-free microcentrifuge tube.
4. 300μl 5 M NaCl and 500μl chloroform were added, and the sample was mixed gently by inverting the tubes six to eight times and centrifuge the sample at 4°C for 5 min at 13000×g to separate the phases. The aqueous phase was transferred (~600μl) to a new 1.5 ml RNase-free microcentrifuge tube.
5. An equal volume of a mix of phenol:chloroform (1:1) was added to the aqueous phase, which was then mixed gently by inverting the tubes six to eight times. Then, the sample was centrifuged at 4°C for 5 min at 13000×g to separate the phases.
6. The upper aqueous layer (~400μl) was carefully transferred to a new 1.5 ml RNase-free microcentrifuge tube, and an equal amount of isopropyl alcohol was added to the tube; then, the mixture was mixed gently by inversion with a swing type vortex mixer (QB-212, Haimen Kylin-Bell Lab Instruments Co., Ltd.) at room temperature for 10 min.
7. The mixture was transferred to an RNase-free spin column and centrifuged at 4°C for 1 min at 13000×g.
8. The flow-through was discarded, a 500μl 75% (v/v) ethanol was added, and then the mixture was centrifuged at 4°C for 1 min at 13000×g.
9. Step 8 was repeated (discarding the flow-through, adding 500μl 75% (v/v) ethanol and then centrifuging at 4°C for 1 min at 13000×g).
10. The flow-through was discarded, and the mixture was centrifuged at 4°C for 1 min at 13000×g to remove the traces of ethanol.
11. The bottom tube was changed with a new 1.5 ml RNase-free microcentrifuge tube, and 30–50μl DEPC-treated water was added to the spin column.
12. After incubating at room temperature for 2 min, the sample was centrifuged at 4°C for 1 min at 13000×g.
13. To increase the RNA yield, the RNA solution was retransferred to the spin column and incubated at room temperature for 2 min.
14. After centrifuging at 4°C for 1 min at 13000×g, the RNA samples were stored at −80°C.

### RNA analysis

In order to verify the RNA integrity, 1μg total RNA was loaded on a standard 1.0% agarose gel, stained with ethidium bromide, and visualized using UV illuminator Red Alpha Innotech (ProteinSimple, CA, USA) [1,11]. The quantity and quality of isolated RNA were assessed by observing the absorbance at 260 and 280 nm. The A_260_/A_280_ ratio was calculated to determine the purity of RNA sample. RIN values were determined on an Agilent 2100 Bioanalyzer using Expert software (Rev. B.02.08.SI648) for plant RNA [6]. RNA samples were successfully reverse transcribed using Invitrogen SuperScript IIReverse Transcriptase (Life Technologies, USA).

### 1st-strand cDNA Synthesis

The SuperScriptIIReverse Transcriptase (Invitrogen, USA) was used to convert total RNA transcripts into 1st-strand cDNA as per the manufacturer’s instructions, utilizing RNase Inhibitor (RNasin, Qiagen). A PCR primer pair that is universally applicable to plants was used for the amplification of the *ALMT1* gene. The sequences were 5'- ATTGATCACGGCAGAGAG-3' for the forward and 5'-GGTGTACTCCATGACGACG -3' for the reverse primer.

A 20μl reaction volume was performed in a 0.2 ml DEPC-treated PCR tube by taking 5μl of RNA, 1μl of 10 mM dNTP Mix, 1μl of anchored oligo-(dT)_18_ (500μg/ml) and a reaction volume filled to 12μl with DEPC water. The contents were briefly centrifuged to collect all components at the bottom, and the tube was placed at 65°C for 5 min. The tube was removed and chilled immediately on ice. The contents of the tube were collected by brief centrifugation, and then 4μl 5×PrimeScript Buffer (250 mM Tris-HCl, pH 8.3, 375 mM KCl, 15 mM MgCl_2_), 2μl 0.1 M DTT, 1μl RNase Inhibitor (Rasin), and 1μl SuperScript Reverse Transcriptase were added to the mixture. The reaction was performed at 42°C for 50 min. The reaction was inactivated by heating at 70°C for 15 min. Subsequently, the tube was removed and quickly chilled on ice. The cDNA could then be used as a template for amplification in PCR.

### PCR Amplification

PCR amplification of first-strand cDNA was performed using Taq DNA polymerase. In this experiment, only 10% of the first-strand reaction products were used for PCR. Higher volumes may not increase amplification and may result in decreased amounts of PCR product. Therefore, the reaction was made by using 5μl of 10×PCR buffer (200 mM Tris-HCl, pH 8.4, 500 mM KCl), 1.5μl of 50 mM MgCl_2_, 1μl of 10 mM dNTP Mix, 1μl of 10μM forward primers, 1μl of 10μM reverse primers, 0.4μl of 5 U/μl Taq DNA polymerase, 2μl cDNA template, and a reaction volume filled to 50μl with DEPC water. The PCR cycles were programmed as follows: initial denaturation at 94°C for 2 min followed by identical 40 cycles with 1 min denaturing at94°C, 1 min annealing at 52°C, and 1 min elongation at 72°C followed by a single final cycle of 5 min at 72°C. After the reaction, 10μl of assay mixture was used for agarose electrophoresis using TAE buffer (1×) following the usual procedure. The remaining products were stored at -20°C.

## Results and Discussion

Methods for RNA extraction are usually evaluated in terms of quantity, quality, and integrity for RT-PCR, qPCR, cDNA library construction, and expression analysis with a proper representation of each expressed gene. However, Chinese fir is often rich in polyphenolics, polysaccharides, proteins, lipids, and several other secondary metabolites that interfere with RNA extraction by degrading or coprecipitating with RNA. For this particular reason, RNA isolation from Chinese fir tissues containing high levels of extractable polyphenolics [12], proteins, lipids [13] and other secondary metabolites [14] is very difficult. Therefore, RNA isolation methods developed for Chinese fir tissues often require substantial modifications [15]. Several total RNA extraction methods including commercial reagents were used to successfully isolate high-quality total RNA of Chinese fir tissues. These methods, however, are subject to important limitations: many reports utilize expensive commercial extraction kits [8,16–22], and others follow tedious procedures utilizing traditional CTAB or modified CTAB extraction methods (up to 2 days) [23–25].

In addition, using the RNA isolation kit to extract the total RNA of Chinese fir tissues is expensive. Therefore, it is necessary to find an efficient, rapid, and reproducible method for isolating high-quality RNA from Chinese fir tissues. The current study describes a rapid, efficient and reliable method that allows the isolation of high-quality total RNA from Chinese fir tissues. The RNA prepared by this protocol was verified for high quality and quantity, and it was successfully used for downstream applications such as RT-PCR, qPCR, cDNA library construction, gene expression analysis, and RNA-seq (unpublished data).

The success of total RNA isolation should be verified by testing the quantity, quality and integrity of RNA. The yields of total RNA (μg/g fresh wt.) isolated from the roots and leaves of Chinese fir are shown in Table 1. The yields of total RNA extracted from the roots ranged from 75.6μg/g to 103.82μg/g, whereas that of the leaves were ranged from 111.5μg/g to 165.00μg/g (Table 1). The reason for the higher yields of total RNA isolated from leaves than roots may be the lower water content in leaves.

**Table 1:** Yields and purity of total RNA isolated from Chinese fir roots and leaves using the described method.

Sample name	Concentration (ng/μl)	Volume(μl)	Yield(μg/g)	A_260/280_	A_260/230_	RIN	28S/18S	Tissues
R1L	275.0	30	165.00	2.05	1.82	7.7	1.5	leaf
R2	272.0	30	163.20	2.07	1.97	7.5	1.4	leaf
R3	232.2	30	139.32	2.07	1.90	7.5	1.4	leaf
2	223	50	111.5	2.10	2.05	7.8	1.5	leaf
R1	207.64	50	103.82	2.08	2.10	8.9	1.8	root
RC	207.24	50	103.62	2.03	2.01	8.9	2.1	root
R0-3-4	312.0	30	93.6	2.07	2.18	9.2	2.0	root
R0-1-4	252.0	30	75.6	2.03	2.15	9.1	1.9	root

**Fig 1:**
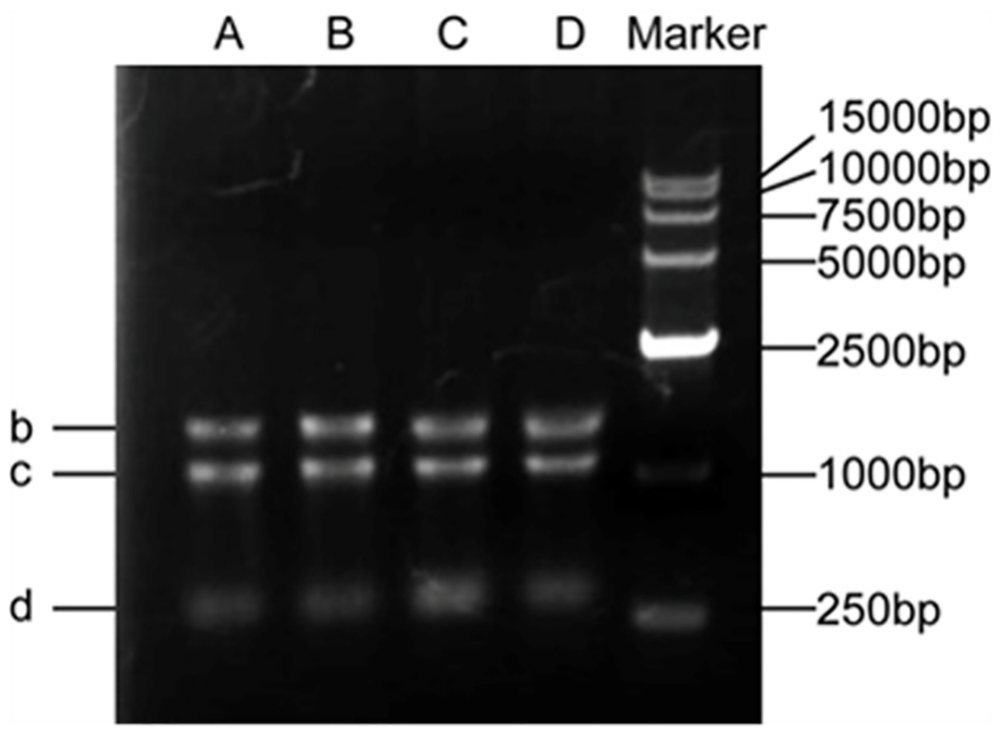
Example of agarose gel electrophoresis of total RNA isolated. Visualization of three intact RNA bands for 28 S RNA (b), 18 S RNA (c) and 5 S RNA (d). Lanes A and B contain 1 μg of total RNA from Chinese fir leaves, and Lanes C and D contain 1 μg of total RNA from Chinese fir roots.

**Fig 2:**
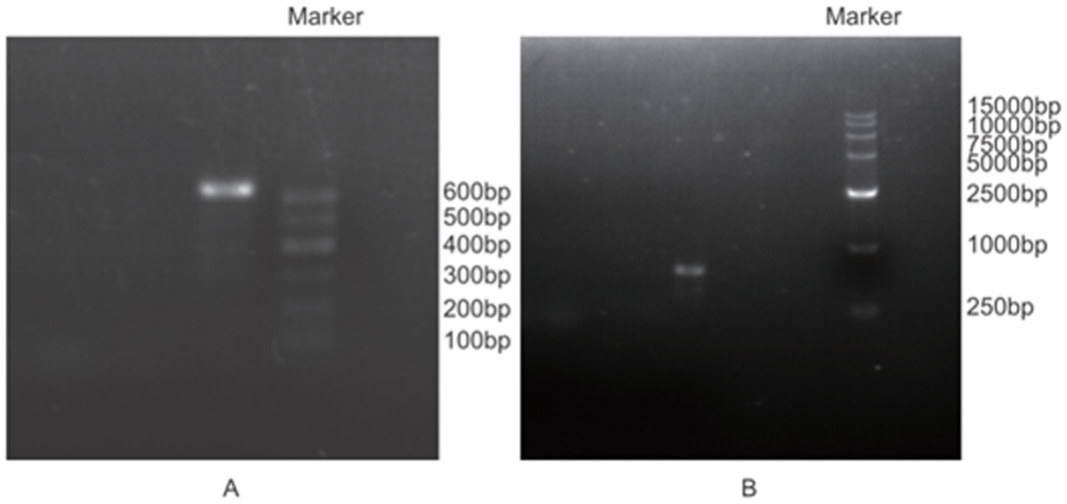
Example of agarose gel electrophoresis of the RT-PCR products. A: leaf, DNA marker (600 bp); B: root, DNA marker (15000 bp).

**Fig 3:**
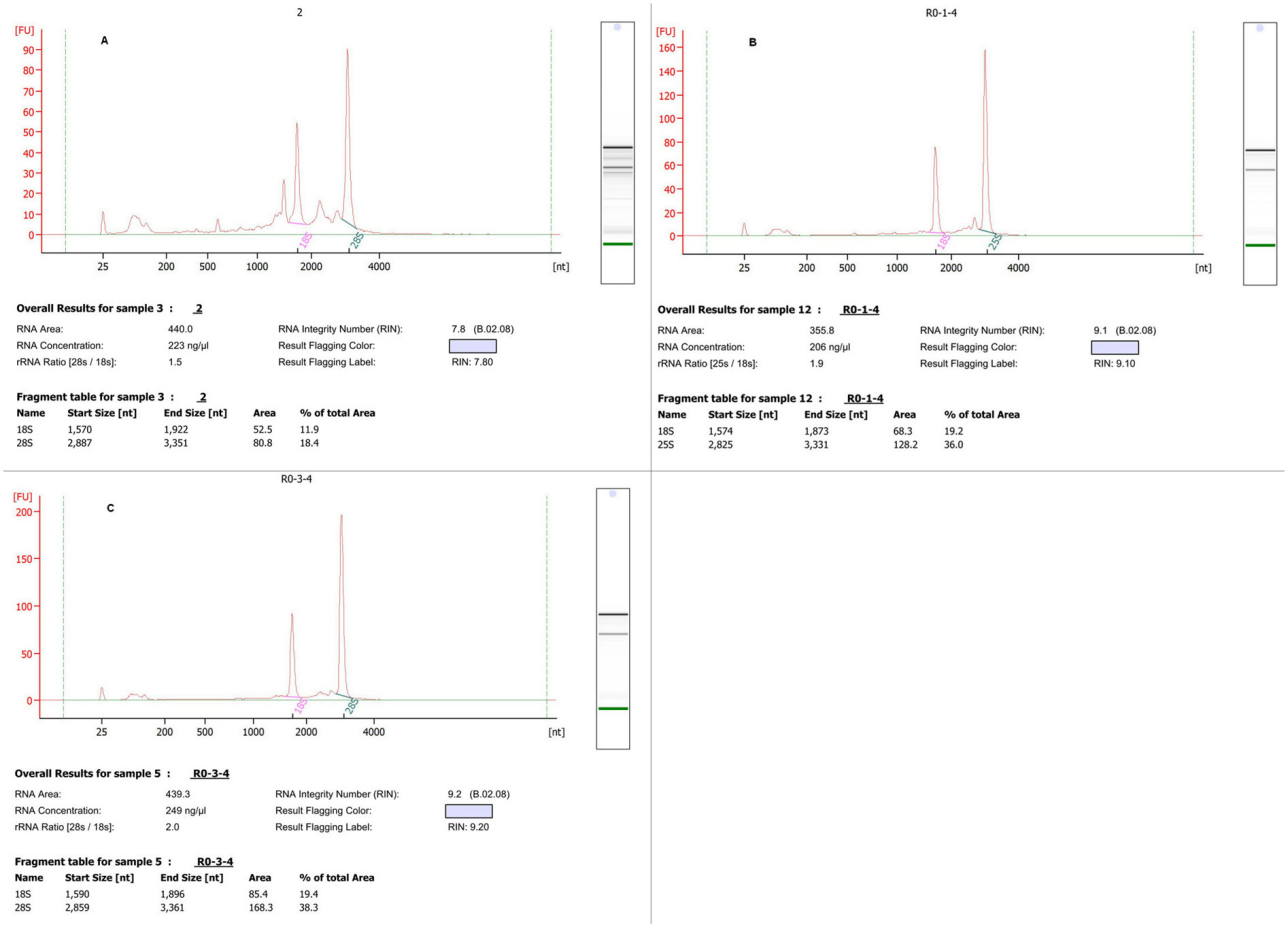
Bioanalyzer results of total RNA isolated using the method described in this study. Isolations were made from three different tissues (leaves and roots) and run on an Agilent 2100 Bioanalyzer (A: leaf; B, C: root). The corresponding virtual gel image generated by the software is depicted on the right side of the electropherogram. The Plant RIN value assigned by the 2100 expert software (Rev. B.02.08.SI648) is indicated. The y axis represents fluorescence units (FU), and the x axis represents seconds (s).

The quality of isolated total RNA described in this study was confirmed in several ways. **Firstly**, 1.0% agarose gel electrophoresis showed that ribosomal bands of 28 S (b), 18 S (c) and 5 S (d) were intact and bright, demonstrating that the total RNA isolated with our detailed method were not degraded, and no high molecular weight gDNA contamination was detected (Fig 1 and S1 Fig). In addition, the ratio of 28 S and 18 S ranged from 1.4 to 2.1, indicating good RNA quality (Table 1). **Secondly**, spectrophotometer analysis showed that the A_260/280_ ratios of RNA samples ranged between 1.9 and 2.1 (Table 1), indicating that the RNA extracted by this approach was largely free of contaminating proteins, DNA, polyphenolics, and polysaccharides. In addition, the A_260/230_ values were ranged from 1.82 to 2.18 (Table 1), indicating that RNA was of high purity and without contamination by organic solvents, secondary metabolites, polyphenolics and polysaccharides [4]. **Thirdly**, the quality of total RNA was assessed using RT-PCR. RNA samples were successfully reverse transcribed using Invitrogen SuperScript IIReverse Transcriptase (Life Technologies, USA), and the target gene was amplified from cDNA by PCR (Fig 2). As indicated in Fig 2, the PCR products were checked for the presence of the objective band on 1% agarose gel. PCR products for detecting the *ALMT1* gene exhibit the target band with the expected size of approximately 600 bp (Fig 2). **Last but perhaps most importantly**, an electropherogram of the total RNA isolated from Chinese fir leaves and roots tissues was generated after analysis with the Agilent 2100 Bioanalyzer. A representative electropherogram of total RNA and the generated gel images showed clear peaks of rRNAs and intact RNA of high quality (Fig 3). In general, RNA with a value above 7.0 is required to produce good results in a next-generation sequencing analysis [4]. The RIN values are shown for three samples isolated with our detailed method (Table 1 and Fig 3). We obtained the total RNA of leaf and root tissues of Chinese fir with RINs of 7.8 (Fig 3A), 9.1 (Fig 3B) and 9.2 (Fig 3C), respectively, indicating no degradation of the RNA, and the Bioanalyzer results of the residual samples were addressed in supplement files (S2 Fig, S3 Fig, S4 Fig, S5 Fig and S6 Fig). These RIN values are much higher than those in previously published reports [20]. The high-integrity RNA obtained from the different Chinese fir tissues used in the current method was suitable for RT-PCR and construction of cDNA libraries.

Significant modifications in this method involve adding 300μl 5 M NaCl and 500μl chloroform to the tube that contained 600μl supernatant, and the ratio of these must be strictly controlled. An excess of chloroform improved neither the RNA quality nor the RNA yield. Moreover, a high concentration of NaCl could effectively separate the contaminants in plant tissues [7]. NaCl was reported to play an important role in nucleic acid isolation from polysaccharides [26]. In addition, high concentrations of EDTA and β-mercaptoethanol were used for inhibiting the oxidization of polyphenol to polyquinones and RNase activity as well [27]. To shorten the time of the whole extraction process, spin columns were used in our protocol. The current protocol can be carried out within approximately two hours by a moderately experienced researcher, and many samples can be processed at the same time, in comparison with 6 hours or the overnight incubation needed in the method of CTAB. Low amounts of reagents used throughout the whole extraction process contribute to a further lowering of costs. The price of isolating the total RNA of one sample with the fresh weight of 0.1 g is 2 RMB, approximately 15 times lower than commercial kits (RNAprep Pure Plant Kit (TIANGEN Biotech, BeiJing): 29.6 RMB/sample)(S1 Table).

## Conclusions

This method can extract a high yield of pure, high-quality RNA from Chinese fir leaves and roots tissues, as indicated by the bioanalyzer results as well as the successful downstream use of isolated RNA. The RNA obtained using this method can be used for various downstream applications including RNA-seq, RT-PCR and qPCR(unpublished data). We have successfully used isolated RNA to generate high-quality RNA-seq libraries for mRNA (unpublished data).

In conclusion, the method reported here is useful for RNA extraction from Chinese fir tissues rich in lipids, polyphenolics and other secondary metabolites.

## Supporting Information

S1 Fig Example of agarose gel electrophoresis of total RNA isolated (Lanes A, B and C contain 1 μg of total RNA from Chinese fir leaves) (A: R1L; B: R2; C:R3). (TIF)

S2 Fig Bioanalyzer results of total RNA isolated using the method described in this study (R1L). (TIF)

S3 Fig Bioanalyzer results of total RNA isolated using the method described in this study (R2). (TIF)

S4 Fig Bioanalyzer results of total RNA isolated using the method described in this study (R3). (TIF)

S5 Fig Bioanalyzer results of total RNA isolated using the method described in this study (RC). (TIF)

S6 Fig Bioanalyzer results of total RNA isolated using the method described in this study (R1). (TIF)

S1 Table Price calculate of extracting one sample of Chinese fir by using the method described in the paper. (XLSX)
