# Supplementary figures and images for: Isolation of High-Quality Total RNA from Chinese Fir (Cunninghamia lanceolata (Lamb.) Hook)
Source: PLoS One. 2015 Jun 17;10(6):e0130234. doi: 10.1371/journal.pone.0130234 (PMC4470689; doi:10.1371/journal.pone.0130234)

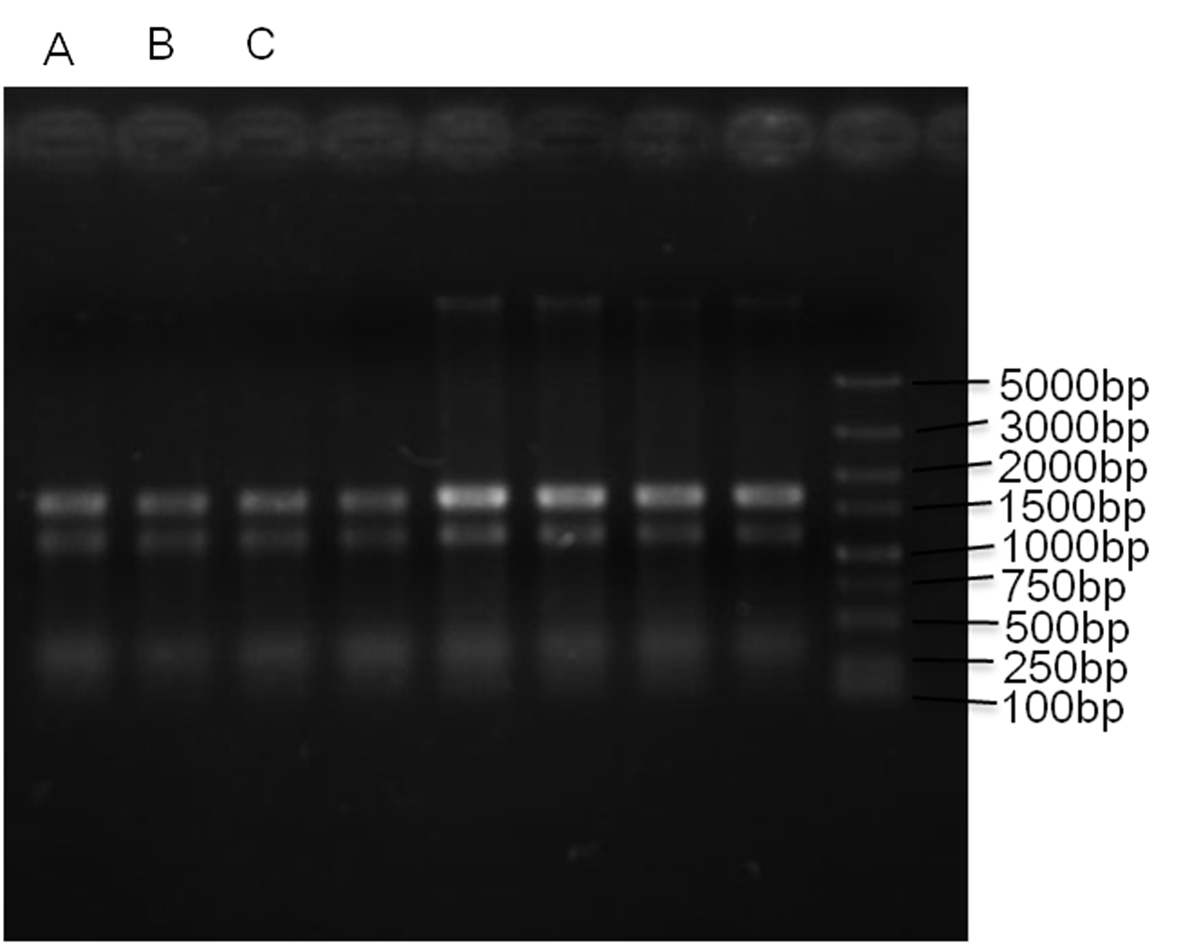

Supplement: S1 Fig — (TIF) [file pone.0130234.s001.tif]

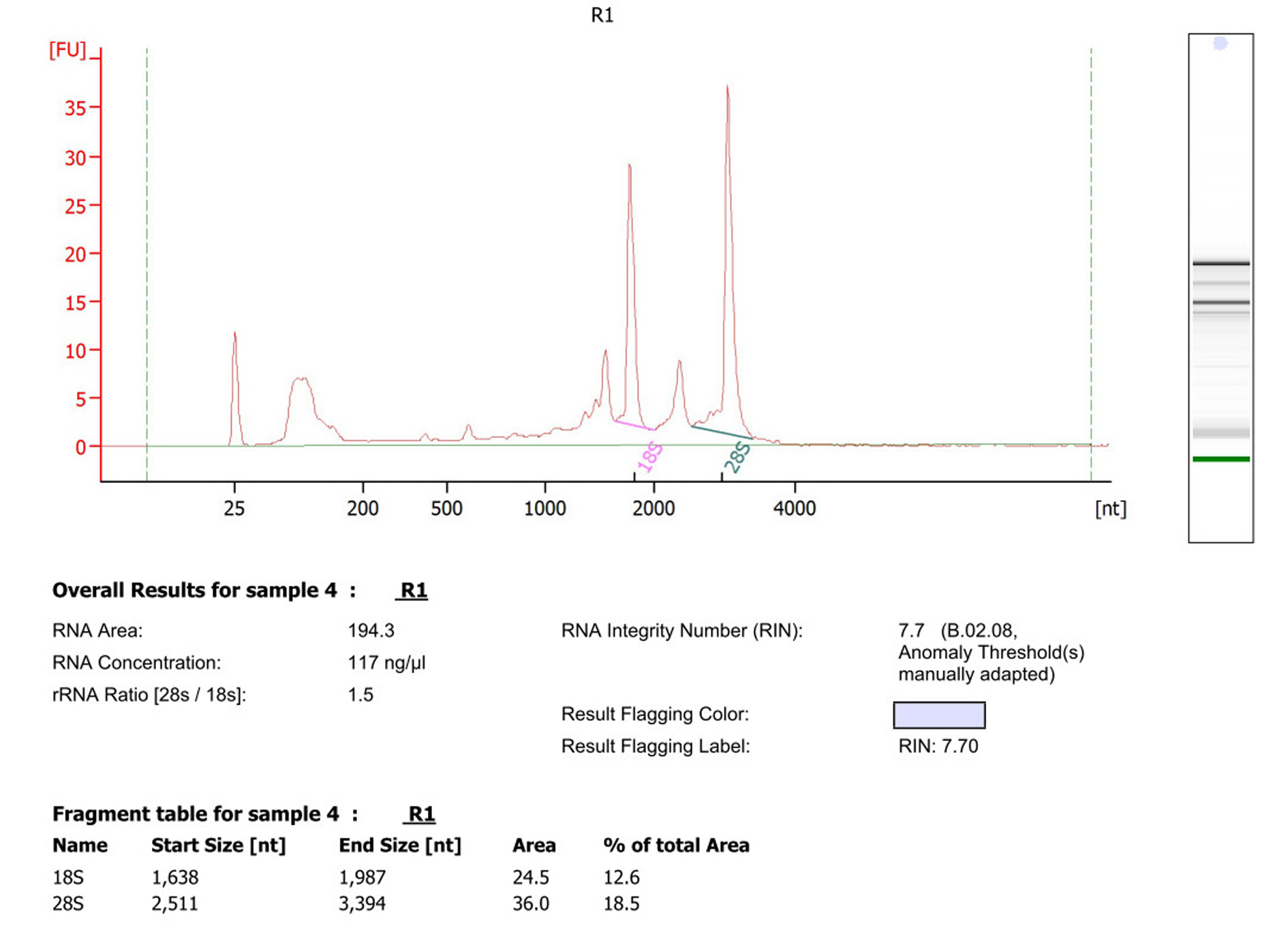

Supplement: S2 Fig — (TIF) [file pone.0130234.s002.tif]

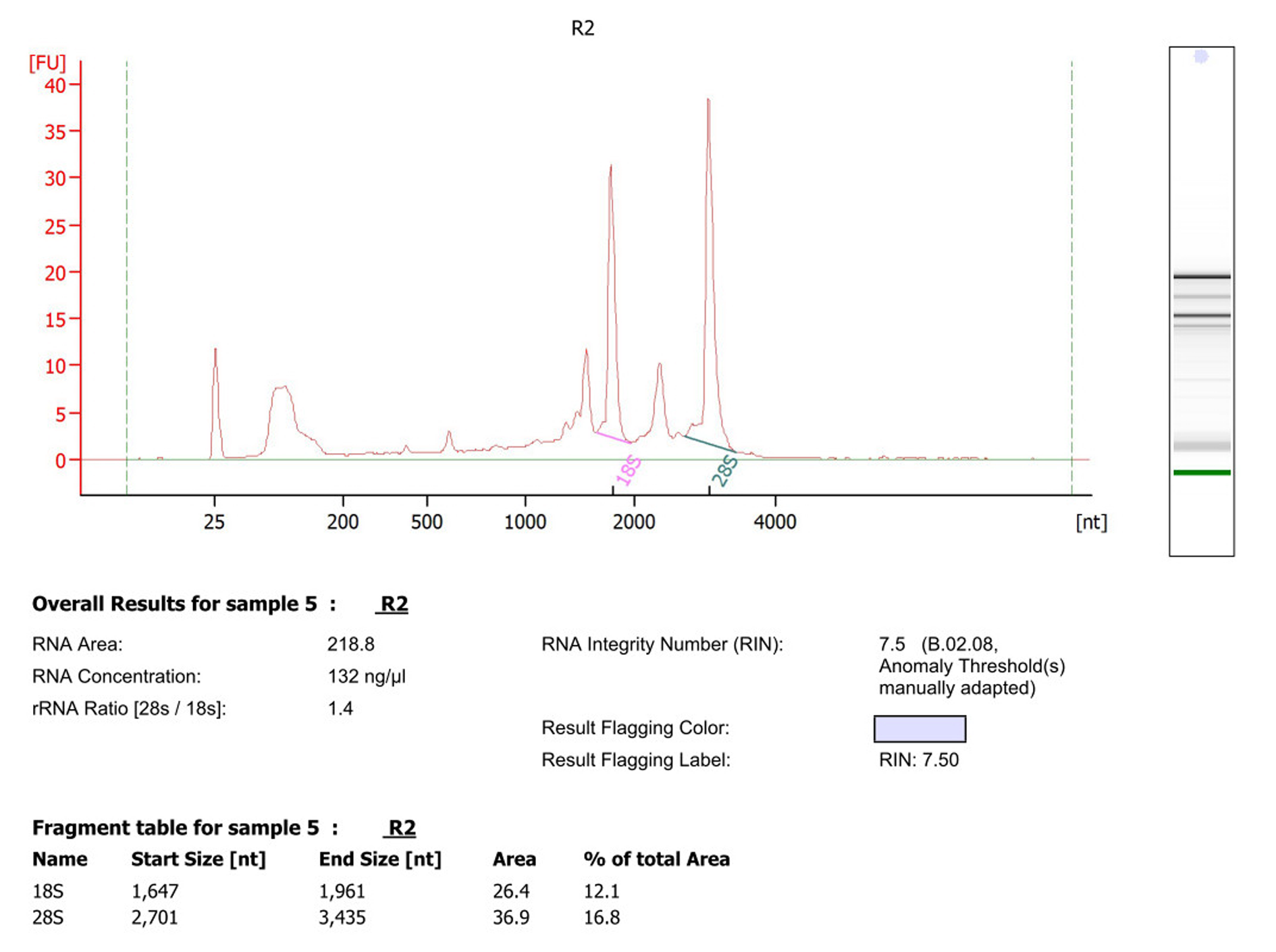

Supplement: S3 Fig — (TIF) [file pone.0130234.s003.tif]

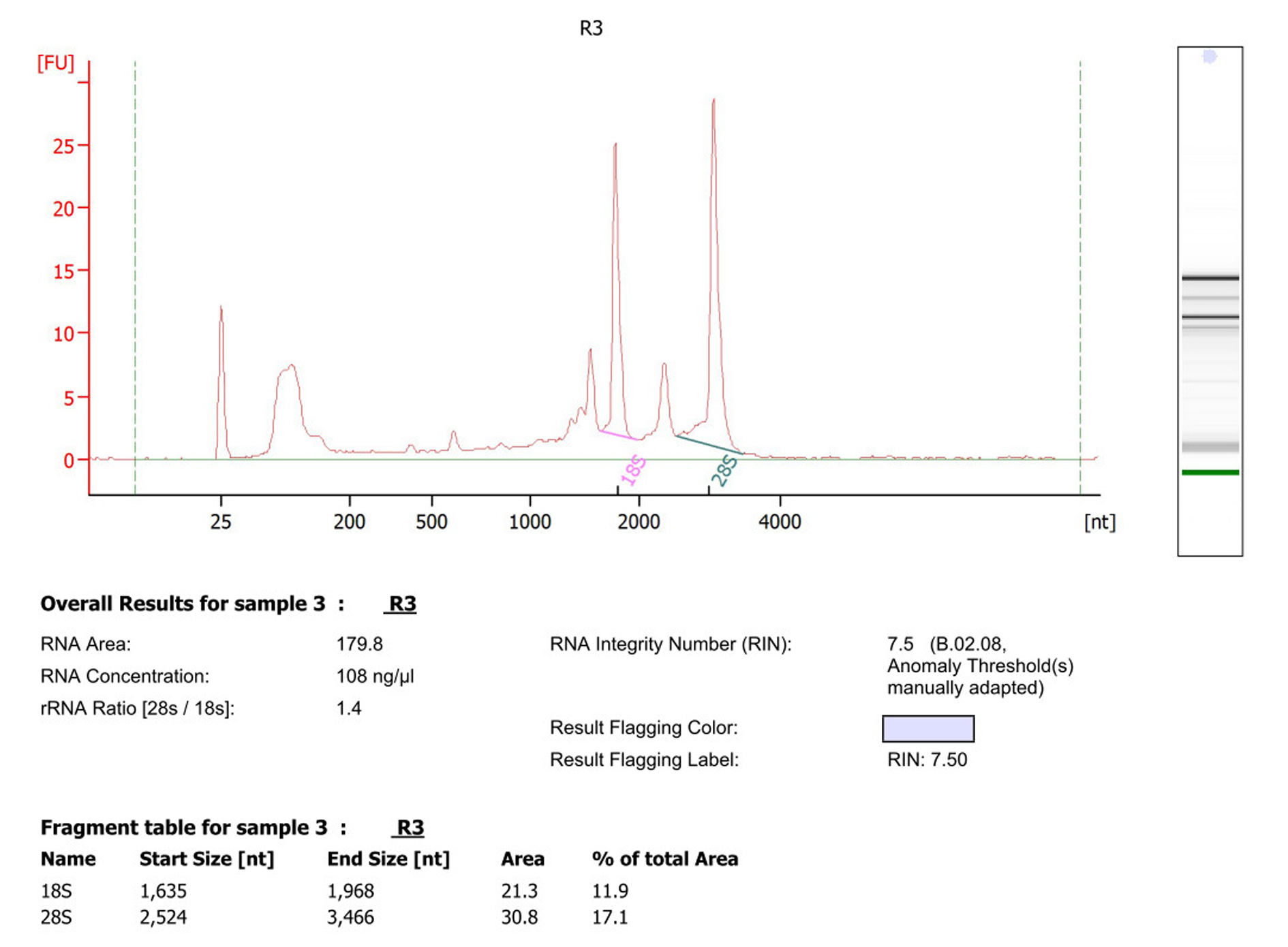

Supplement: S4 Fig — (TIF) [file pone.0130234.s004.tif]

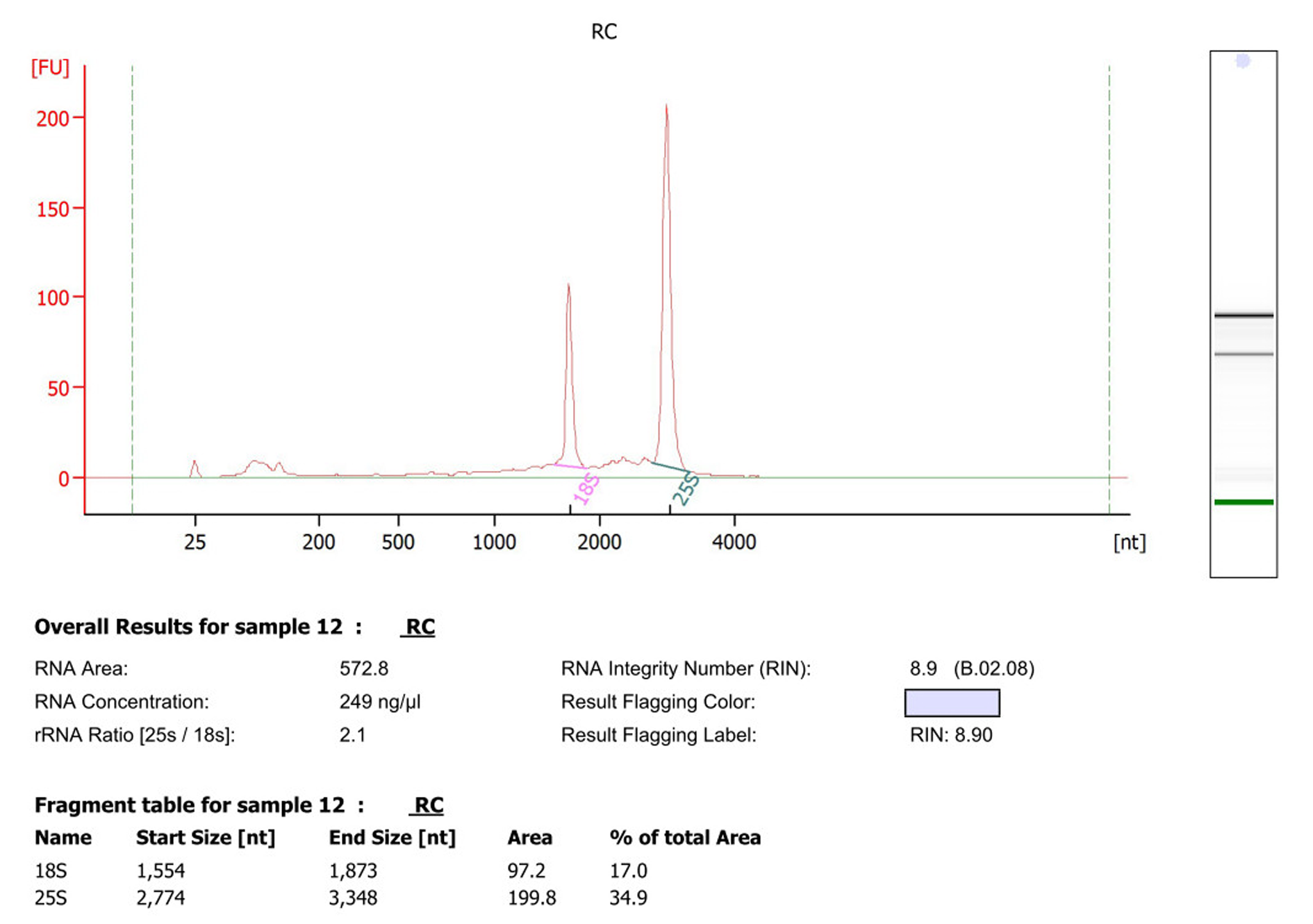

Supplement: S5 Fig — (TIF) [file pone.0130234.s005.tif]

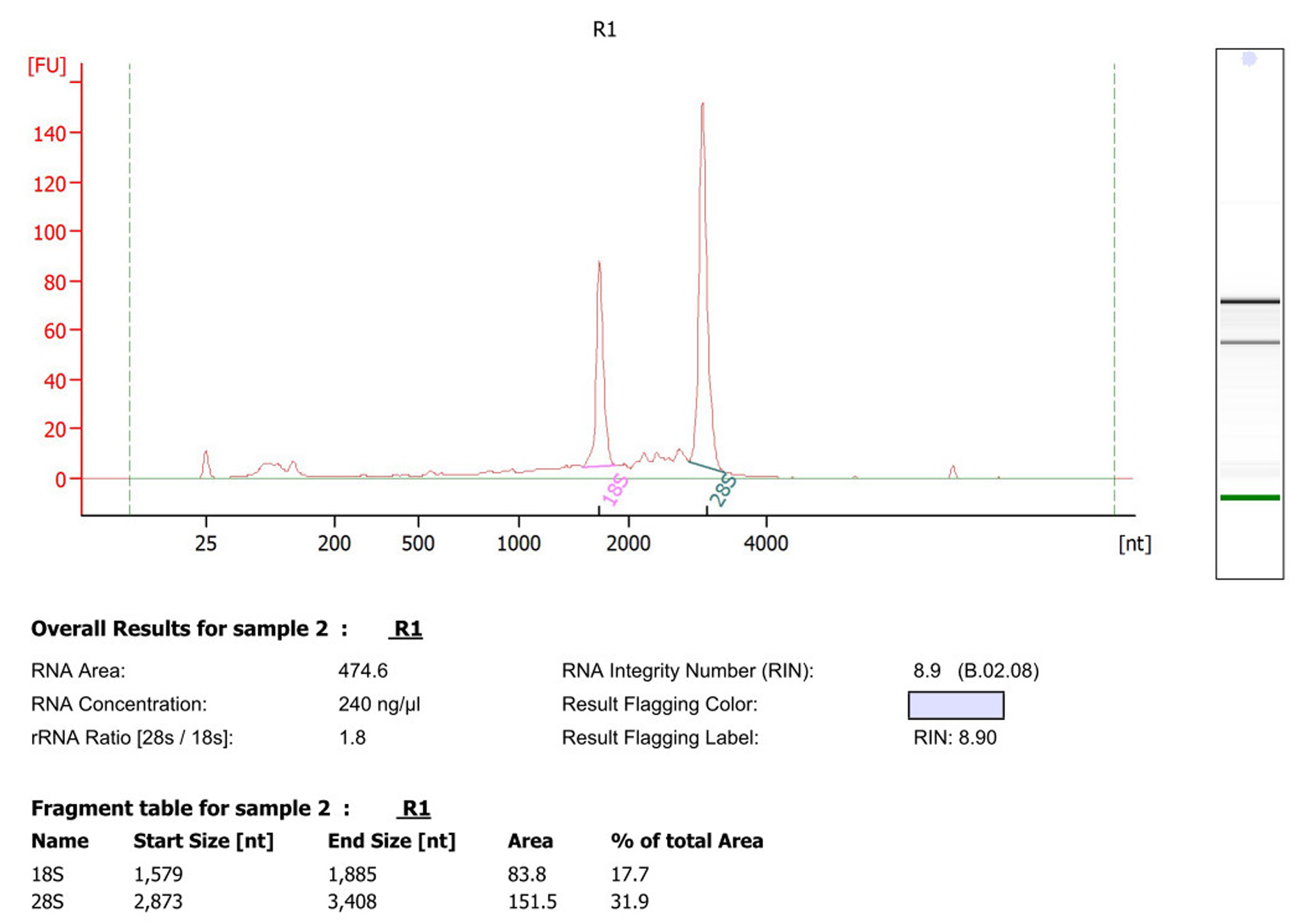

Supplement: S6 Fig — (TIF) [file pone.0130234.s006.tif]
